# Breastfeeding Recommendations: Torn Between Evidence and Emotions—A Commentary

**DOI:** 10.3390/nu18101498

**Published:** 2026-05-08

**Authors:** Berthold V. Koletzko, Frank Jochum, Martina Kohl-Sobania, Walter A. Mihatsch, Diana Rubin

**Affiliations:** 1 Dr. von Hauner Children’s Hospital, LMU University Hospital, Ludwig Maximilians Universität Munich, Lindwurmstrasse 4, 80337 Munich, Germany; 2 German Center for Child and Adolescent Health, 80337 Munich, Germany; 3 Evangelisches Waldkrankenhaus Spandau, 13589 Berlin, Germany; frank.jochum@pgdiakonie.de; 4 Pediatric Medicine, Brandenburg Medical School (MHB), 16816 Neuruppin, Germany; 5 Department of Pediatrics, University Hospital Schleswig-Holstein, Campus Lübeck, 23538 Lübeck, Germany; martina.kohl-sobania@uksh.de; 6 Faculty of Health Management, Neu-Ulm University of Applied Sciences, 89231 Neu Ulm, Germany; walter.mihatsch@uni-ulm.de; 7 Center for Nutritional Medicine and Diabetology, Vivantes Klinikum Berlin-Reinickendorf and Berlin-Spandau, 13509 Berlin, Germany; diana.rubin@vivantes.de

**Keywords:** breastfeeding, complementary feeding, evidence-based medicine, evidence gap, implicit bias

## Abstract

The recently published German evidence-based guideline on “Breastfeeding Duration” recommends that infants in Germany should be exclusively or predominantly breastfed for 6 months, with a total breastfeeding duration of 12 months. These recommendations rely exclusively on low- and very low-certainty evidence, and fall short of what would be expected for an evidence-based guideline. The guideline has been criticized by a large number of eight professional organizations, which is very unusual. As delegates or deputy delegates of our respective medical-scientific societies participating in the six-year guideline development process, we disagree with the published guideline recommendations and present here our key methodological and content-related concerns. In our view, there is no sufficient evidence that would demonstrate superiority of the proposed recommendation with respect to benefits and harms compared to the long-standing recommendation of exclusive breastfeeding for 4–6 months and continued breastfeeding for as long as mother and child desire for healthy populations in Germany and Western Europe.

## 1. Introduction

Breastfeeding is the natural form of infant feeding and is associated with numerous benefits for both child and mother [1]. Longer duration of breastfeeding and higher amounts of milk delivered are associated with greater degrees of benefits for health outcomes such as moderate-to-severe respiratory and gastrointestinal infections, otitis media, allergic rhinitis, asthma, inflammatory bowel disease, type 1 diabetes, rapid weight gain and obesity, and high systolic blood pressure [2]. Therefore, physicians and other healthcare professionals are dedicated to actively promoting, protecting, and supporting breastfeeding [3].

In Germany, previously established and broadly consented recommendations for healthy infants advise exclusive breastfeeding (i.e., only breast milk, but no other liquids such as water or tea, no solid food, with the only exceptions being vitamins, minerals or medications) for the first 4–6 months of life, introduction of complementary foods between 17 and 26 weeks, and continued breastfeeding as long as mother and child wish [4,5,6]. These recommendations align with those of European and American professional societies [7,8,9] and with most national recommendations across Europe [10]. Until 2001, the World Health Organization (WHO) issued similar guidance, but since then has issued a worldwide recommendation for exclusive breastfeeding for 6 months, with complementary feeding beginning only in the seventh month, and continued breastfeeding up to 2 years or beyond [11,12]. This recommendation is grounded primarily in the associated protection against infection-related morbidity and mortality in low- and lower-middle-income countries [12,13]. However, for conditions such as those found in Western Europe, corresponding data are not available. Divergence of national, pediatric, and WHO recommendations has fueled controversy for many years. Consequently, three German professional societies (German Society for Pediatrics and Adolescent Medicine, German Society for Gynecology and Obstetrics, and German Society for Midwifery Science) registered the development of an evidence-based guideline (considered as an “S3 guideline” based on the classification of the German Working Group of Medical Scientific Societies, AWMF) [14] on “Breastfeeding Duration and Breastfeeding Interventions.” Coordinated by the pediatric nutrition division of the Max Rubner Institute (MRI), a German Federal Government research institute, work began in February 2020. After six years, the first part—addressing breastfeeding duration—was published in February 2026 [15]. Work on the second part of the guideline addressing breastfeeding interventions is ongoing. The guideline recommended that infants should be breastfed exclusively or predominantly (i.e., only breast milk and other liquids such as water or tea, but no nutrient-rich liquids, solid or other complementary foods) for 6 months, and breastfeeding should continue at least until 12 months (Table 1). The authors of this paper served as delegates or deputy delegates of our medical societies in the process of guideline development, but do not support the resulting recommendations. Here, we summarize our concerns.

## 2. Critical Evaluation

### 2.1. Substantial Professional Opposition

Unusual for an evidence-based guideline, as many as eight professional organizations raised methodological and substantive objections [16,17]. Four organizations issued formal dissenting votes opposing the recommendation on total breastfeeding duration (German Society for Pediatric Dentistry, German Society for Social Pediatrics and Adolescent Medicine) or both recommendations (German Society for Nutritional Medicine, Professional Association of Primary Care Paediatricians). These dissenting votes are reproduced in the final guideline. Three additional organizations (German Society for Pediatrics and Adolescent Medicine, Society of Pediatric Allergology and Environmental Medicine, Directorate of the Max Rubner Institute, where the guideline development was coordinated) submitted written concerns without formal dissent, which therefore are not included in the guideline with named organizations. Following the publication of the guideline, the Society for Pediatric Gastroenterology and Nutrition, with members mostly from Germany, Austria, and Switzerland, who had not been invited to participate in the guideline development, also rejected the recommendations [16].

The guideline text rightfully states that breastfeeding promotion and counseling are most effective when all professional groups consistently communicate essential information [11]. Obviously, this important goal was not achieved. Broad implementation of the guideline recommendations is hardly possible given the rejection of both recommendations by the Professional Association of Primary Care Paediatricians (BVKJ), whose members are among the most influential advisors to families on infant nutrition issues.

### 2.2. Methodological Critique

#### 2.2.1. Formulation of PECO Questions

According to the established standards for medical guidelines, these should weigh the benefits and harms of alternative approaches based on systematic evidence assessment [14]. For the guideline in question, this would have required evaluating the evidence supporting the hitherto established German recommendations versus the alternative WHO recommendations. Accordingly, appropriately formulated PECO questions (population, exposure, control, and outcome), which serve to direct the evidence analysis of the guideline, would have compared the proposed alternative recommendations, i.e., the WHO recommendations on breastfeeding duration as “exposure” versus the established recommendations as “control”. In contrast, here the PECO questions were worded to only explore positive or negative health effects of the WHO recommendation (Table 2). This choice prevented an unbiased comparison of the benefits and disadvantages of the different options and suggests that the guideline coordinators were biased towards the WHO recommendations.

#### 2.2.2. Weaknesses in the Evidence Base

The evidence assessment relied exclusively on observational studies reporting associations, with low or very low-certainty evidence, which does not meet the standards expected for an evidence-based medical guideline. Delegates’ proposals to include the available high-certainty evidence from several randomized controlled trials comparing different times of introducing complementary feeding, resulting in different durations of exclusive breastfeeding, were rejected.

A large part of the available observational studies on breastfeeding inadequately adjust for confounders such as socioeconomic status, household smoking, timing of complementary feeding, or daycare attendance [2]. Definitions of breastfeeding exposure are heterogeneous and often unreliable. There is a lack of data that allows comparison of different, defined durations of breastfeeding or of exclusive or predominant breastfeeding.

Breastfeeding duration was mostly assessed based on maternal reporting at single or a few time points, in many studies using retrospective data collection with high error risks due to recall bias, where occasional provision of other feeds can easily be overlooked. There is also an understandable tendency towards socially desirable answers. Maternal recall of exclusive breastfeeding was shown to substantially overestimate objectively measured exclusive breastfeeding with the isotope-based Deuterium Dose-to-Mother technique by 40% [18] or even by a 2–5-fold overestimation [19,20]. Thus, estimation of exclusive breastfeeding based on maternal interviews is rather unreliable. However, the evidence from observational studies on which the guideline and its recommendations are based relies heavily on these subjective reports on breastfeeding behavior with low reliability, which limits the validity of possible conclusions.

Overall, the guideline recommendations are based on weak data from observational studies with low or very low-certainty evidence; therefore, causal conclusions cannot be drawn [2,21,22]. There are no health endpoints for which a clear advantage for a specific duration or intensity of breastfeeding was demonstrated [2]. The strong recommendation for a minimum total breastfeeding duration of 12 months (Table 1) based only on very low or low certainty of evidence is not justified if one applies the criteria of the Core GRADE system (Grading of Recommendations Assessment, Development and Evaluation) [23].

#### 2.2.3. Selection and Prioritization of Endpoints

Initially, a rather large number of 21 child and nine maternal health outcomes were determined for inclusion in the evidence analysis [17], including initially also the clinically irrelevant endpoint left-handedness (for which a risk reduction by breastfeeding was determined!). Clinically relevant outcomes, including micronutrient status, food allergies, and effects on maternal mental health, were excluded despite delegate requests. The national standards for guideline development call for determining clinically relevant endpoints on which evidence-based conclusions are based, early in the guideline development process [14]. In contrast, in this guideline, the nine endpoints on which recommendations were based were selected late in the process. While a scoring system was used, the choices were based on subjective judgments of guideline group members [17]. Outcomes showing the strongest positive effects in the evidence analysis were selected. This constitutes post hoc, biased selection and is methodologically unacceptable.

### 2.3. Content-Related Critique

#### 2.3.1. General Evidence

A recent high-quality systematic review, including 29 systematic reviews and 145 original studies on breastfeeding and health endpoints, found that more breastfeeding is associated with a reduced risk for several health outcomes [2]. However, the authors concluded there was no evidence to justify recommending a specific duration (e.g., 4, 6, or 12 months) of either exclusive, predominant, or any breastfeeding.

#### 2.3.2. Infectious Diseases

A recent WHO-commissioned review of studies performed worldwide found little or no effect of exclusive breastfeeding duration or timing of complementary feeding on diarrhea, respiratory infections, growth, obesity, or anemia [12]. The German guideline reports low-certainty evidence for an association of reduced otitis media and gastrointestinal infections with longer duration of exclusive breastfeeding. In contrast, the results of a large Norwegian cohort study with about 70,000 infants showed no increase in serious infections leading to hospital treatment with earlier introduction of complementary feeding and hence shorter duration of exclusive breastfeeding [24]. Likewise, a systematic review of the European Food Safety Authority (EFSA) found no infection-protective effect of longer exclusive breastfeeding in European infants, that usually enjoy good hygienic conditions [25]. At a low incidence of feverish infections among Western and Central European infants, breastfed and non-breastfed infants do not show a different infection rate [26].

#### 2.3.3. Asthma

The guideline concludes that exclusive breastfeeding for 3–6 months, or for 6 months, is associated with less childhood asthma than shorter or no breastfeeding [15]. A recent systematic review found observational studies with considerable methodological limitations, suggesting reduced early wheezing with longer breastfeeding, but inconclusive evidence for effects on asthma at later ages, with the data failing to provide a basis for recommending a specific breastfeeding duration [2].

#### 2.3.4. Micronutrient Status

Despite delegate requests, micronutrient status was excluded as an endpoint in the guideline [15]. The evidence analysis underpinning the WHO breastfeeding recommendation of 2001 reported indications for possible adverse effects of an exclusive breastfeeding duration for 6 months on infant hematological parameters [11]. In its systematic assessment of the appropriate age for the introduction of complementary foods into an infant’s diet, the European Food Safety Authority (EFSA) pointed out that infants with a risk of iron deficiency benefit from earlier introduction of complementary foods before the age of 6 months, without any disadvantages arising [25]. Recent studies confirm an increased risk of iron and zinc deficiency with exclusive breastfeeding during the first 6 months [27,28,29]. In Europe, this is particularly relevant for high-risk groups, such as infants born with low birthweight or born prematurely.

#### 2.3.5. Food Allergy

Despite a high disease burden, food allergy prevention was also excluded as an endpoint in the guideline’s evidence evaluation [15]. Numerous randomized trials show substantial reductions in food allergy risk with early introduction of allergenic foods prior to the age of 6 months [8,30,31]. The randomized controlled PreventADALL trial enrolling almost 2400 infants demonstrated that early introduction of common complementary foods markedly reduced food allergy at age 3 years, with 60% less total food allergies, 70% less cows’ milk allergy, and 60% less peanut allergy [32]. The European EAACI guideline identifies 4–6 months as the most effective age window for introducing peanut products to reduce later food allergy [8]. Of importance, earlier introduction of complementary feeding was shown not to reduce breastfeeding frequency or duration, and not to have adverse effects on growth or nutrient supply [33]. The strong evidence for markedly reducing food allergy risk with earlier complementary feeding introduction provides a compelling argument against recommending exclusive or predominant breastfeeding for 6 months in European populations.

#### 2.3.6. Dental Health

A recent systematic review of 31 studies (28,000 children) found exclusive breastfeeding under 6 months associated with about a halved risk of early childhood caries, whereas breastfeeding for more than 12 months and nighttime breastfeeding were associated with a 2.4–7.1-fold increased caries risk, with no indication that results would be driven by potentially associated sugar intake or nighttime provision of other foods [34]. This led the German Society for Pediatric Dentistry to oppose the strong recommendation for a breastfeeding duration of at least 12 months [15] (Table 1).

#### 2.3.7. Developmental Readiness

Neurodevelopmental readiness for introducing complementary feeding was not addressed in the guideline [15]. Infants show large inter-individual variation. The earliest developmental skills relevant for the consumption of pureed complementary foods can be observed between 3 and 4 months of age; the ability to eat finger foods is observed in some infants as early as 4 months, but more frequently at 5–7 months [25]. The previously established recommendation with a window ranging from 4 to 6 months of age as an appropriate time for introducing complementary feeding allows for responding to the different signals and developmental readiness of individual infants.

#### 2.3.8. Maternal Mental Health

Evidence-based clinical practice guidelines should be based on an evidence-based assessment of the benefits and harms of alternative approaches [14]. Potential harms of the recommendations—infant nutrient deficiencies, allergy risk, and caries—were insufficiently considered in the guideline on breastfeeding duration, as were maternal psychological burdens [15]. Most infants in Germany and worldwide are not exclusively breastfed for 6 months, nor breastfed for 12 months [35,36]. Breastfeeding recommendations that many mothers do not or cannot meet may induce guilt, the feeling of being a “bad mother”, depressive symptoms, and disturbed mother–infant bonding [37,38,39,40,41]. Such possible adverse effects are particularly concerning when respective strong recommendations are made even though a strong evidence base is lacking, as is the case here for the strong recommendation to breastfeed for 12 months, and pertain to preventive—not therapeutic—measures.

## 3. Conclusions

Breastfeeding is the optimal form of infant nutrition, and healthcare professionals should actively promote, protect, and support it. However, uniform global recommendations for breastfeeding duration are inappropriate given the diverse conditions of life and health. For infants in Germany and Europe, current evidence does not justify recommending exclusive or predominant breastfeeding for 6 months, delaying complementary feeding until month 7, or requiring a total breastfeeding duration of at least 12 months. Counseling should encourage breastfeeding for as long as possible and emphasize that any breastfeeding is beneficial, including partial breastfeeding. Early introduction of complementary foods, including allergenic foods, between 4 and 6 months of age can reduce food allergy risk and should be recommended for infants from populations with a low infectious disease burden but a high food allergy risk, provided the infant shows signs of readiness for semisolid feeding. The benefit of continued breastfeeding along with complementary feeding should be emphasized. Screening for iron depletion, e.g., by measurement of hemoglobin and ferritin, should be considered in infants with clinical signs suggestive of iron depletion, and in high-risk groups such as infants born small or premature. Breastfeeding promotion and support is important, however individualized, non-dogmatic counseling on all aspects of maternal, infant and young child nutrition, with a sense of reason, is warranted.

## Figures and Tables

**Table 1:** Recommendations of the German guideline on breastfeeding duration [13], criticized by seven participating professional organizations and the authors of this paper.

Recommendations
1. Full-term infants should be exclusively * or predominantly ** breastfed until the end of their 6th month of life. *(Conditional/weaker recommendation, strong consensus)*
2. The total duration of breastfeeding for full-term infants should be at least 12 months. *(Strong recommendation, consensus)*

* Exclusive breastfeeding: only breast milk, no other liquids (e.g., water, tea), no solid food. Exception: vitamins, minerals, medications. ** Predominant breastfeeding: only breast milk and other liquids such as water/tea, no nutrient-rich liquids/solid food/complementary foods.

**Table 2:** PECO questions guiding the evidence analysis of the German guideline on breastfeeding duration [13], which, in the authors’ view, are biased towards adopting the WHO breastfeeding recommendations (PECO: population, exposure, control, and outcome).

Recommendations
PECO 1: Are there positive or negative health effects for children (P) and mothers (P) when breastfeeding is carried out according to the WHO breastfeeding recommendation (six months exclusively) or predominantly for six months (E)? How do the results compare with a shorter breastfeeding duration (C) or no breastfeeding (C) with regard to various health outcomes (O)?
PECO 2: Are there positive or negative health effects for children (P) and mothers (P) when breastfeeding continues for at least twelve months (total breastfeeding duration, regardless of breastfeeding intensity) (E)? How do the results compare with a shorter total breastfeeding duration (C) or no breastfeeding (C) with regard to various health outcomes (O)?

## Data Availability

No new data were created or analyzed in this study. Data sharing is not applicable to this article.

## References

[1] Prell C., Koletzko B. (2016). Breastfeeding and complementary feeding—Recommendations on infant nutrition. Dtsch. Arztebl. Int..

[2] Patnode C.D., Henrikson N.B., Webber E.M., Blasi P.R., Senger C.A., Guirguis-Blake J.M. (2025). Breastfeeding and Health Outcomes for Infants and Children: A Systematic Review. Pediatrics.

[3] Fewtrell M., Bandsma R.H.J., Baur L., Duggan C.P., Dumrongwongsiri O., Hojsak I., Khatami K., Koletzko B., Kovalskys I., Li Z. (2023). The role of Pediatricians in promoting and supporting breastfeeding. A position paper of the International Pediatric Association (IPA) Strategic Advisory Group on Infant, Child and Adolescent Nutrition. Ann. Nutr. Metab..

[4] Ernährungskommission der Deutschen Gesellschaft für Kinder-und Jugendmedizin (DGKJ) (2014). Ernährung gesunder Säuglinge. Empfehlungen der Ernährungskommission der Deutschen Gesellschaft für Kinder- und Jugendmedizin. Monatsschr. Kinderheilkd..

[5] Koletzko B., Bührer C., Jochum F., Ganschow R., Kauth T., Körner A., Koletzko S., Mihatsch W., Prell C., Ernährungskommission der Deutschen Gesellschaft für Kinder- und Jugendmedizin (DGKJ e.V.) (2016). Zeitpunkt der Beikosteinführung und Risiko für Allergien und Zöliakie. Monatsschr. Kinderheilkd..

[6] Koletzko B., Bauer C.P., Cierpka M., Cremer M., Flothkötter M., Graf C., Heindl I., Hellmers C., Kersting M., Krawinkel M. (2016). Ernährung und Bewegung von Säuglingen und stillenden Frauen. Aktualisierte Handlungsempfehlungen von “Gesund ins Leben–Netzwerk Junge Familie“, eine Initiative von IN FORM. Monatsschr. Kinderheilkd..

[7] European Society for Paediatric Gastroenterology, Hepatology & Nutrition, European Academy of Paediatrics, European Society for Paediatric Research, European Academy for Allergy and Clinical Immunology, Federation of International Societies for Paediatric Gastroenterology Hepatology and Nutrition, Latin American Society for Pediatric Gastroenterology Hepatology and Nutrition, Pan Arab Society for Pediatric Gastroenterology and Nutrition, Asian Pan-Pacific Society for Pediatric Gastroenterology, Hepatology and Nutrition, North American Society for Pediatric Gastroenterology, Hepatology and Nutrition, World Allergy Organization (2024). World Health Organization (WHO) guideline on the complementary feeding of infants and young children aged 6–23 months 2023: A multisociety response. J. Pediatr. Gastroenterol. Nutr..

[8] Halken S., Muraro A., de Silva D., Khaleva E., Angier E., Arasi S., Arshad H., Bahnson H.T., Beyer K., Boyle R. (2021). EAACI guideline: Preventing the development of food allergy in infants and young children (2020 update). Pediatr. Allergy Immunol..

[9] Kleinman R.E. (2000). American Academy of Pediatrics recommendations for complementary feeding. Pediatrics.

[10] Koletzko B., Hirsch N.L., Jewell J.M., Dos Santos Q., Breda J., Fewtrell M., Weber M.W. (2020). National Recommendations for Infant and Young Child Feeding in the World Health Organization European Region. J. Pediatr. Gastroenterol. Nutr..

[11] World Health Organization (2001). The Optimal Duration of Exclusive Breastfeeding. Report of an Expert Consultation.

[12] World Health Organization (2023). WHO Guideline for Complementary Feeding of Infants and Young Children 6–23 Months of Age.

[13] World Health Organization (2003). Global Strategy for Infant and Young Child Feeding.

[14] Arbeitsgemeinschaft der Medizinisch Wissenschaftlichen Fachgesellschaften (2020). Das AWMF-Regelwerk Leitlinien.

[15] Deutsche Gesellschaft für Kinder- und Jugendmedizin e.V., Deutsche Gesellschaft für Gynäkologie- und Geburtshilfe e.V., Deutsche Gesellschaft für Hebammenwissenschaft e.V. AWMF S3-Leitlinie Stilldauer und Interventionen zur Stillförderung. Register Nr. 027/072 2026. https://register.awmf.org/de/leitlinien/detail/027-072.

[16] Gesellschaft für- Pädiatrische Gastroenterologie und Ernährung-(GPGE) (2026). Stellungnahme der Gesellschaft für Pädiatrische Gastroenterologie und Ernährung (GPGE) e.V. zur S3-Leitlinie “Stilldauer und Interventionen zur Stillförderung“ der AWMF.

[17] Deutsche Gesellschaft für Kinder- und Jugendmedizin e.V., Deutsche Gesellschaft für Gynäkologie- und Geburtshilfe e.V., Deutsche Gesellschaft für Hebammenwissenschaft e.V (2026). AWMF S3-Leitlinie Stilldauer und Interventionen zur Stillförderung.

[18] Owino V.O., Slater C., Loechl C.U. (2017). Using stable isotope techniques in nutrition assessments and tracking of global targets post-2015. Proc. Nutr. Soc..

[19] Mulol H., Coutsoudis A. (2018). Limitations of maternal recall for measuring exclusive breastfeeding rates in South African mothers. Int. Breastfeed. J..

[20] Mulol H., Coutsoudis A., Hounkpatin W.A., Urio E., Wabolou P.K., Sissinto Y., El-Kari K. (2020). Is exclusive breastfeeding an option or a necessity in Africa? A pooled study using the deuterium oxide dose-to-mother technique. J. Public Health Afr..

[21] van Rossum C.T.M., Büchner F.L., Hoekstra J. (2005). Quantification of Health Effects of Breastfeeding.

[22] U.S. Department of Agriculture, Agicultural Research Service (2020). Dietary Guidelines Advisory Committee US Department of Agriculture. 2020. Scientific Report, Dietary Guidelines Advisory Committee.

[23] Guyatt G., Vandvik P.O., Iorio A., Agarwal A., Yao L., Eachempati P., Zeng L., Chu D.K., D’Souza R., Agoritsas T. (2025). Core GRADE 7: Principles for moving from evidence to recommendations and decisions. BMJ.

[24] Stordal K., Lundeby K.M., Brantsæter A.L., Haugen M., Nakstad B., Lund-Blix N.A., Stene L.C. (2017). Breast-feeding and Infant Hospitalization for Infections: Large Cohort and Sibling Analysis. J. Pediatr. Gastroenterol. Nutr..

[25] Castenmiller J., de Henauw S., Hirsch-Ernst K.-I., Kearney J., Knutsen H.K., Maciuk A., Mangelsdorf I., McArdle H.J., Naska A., EFSA Panel on Nutrition, Novel Foods and Food Allergens (NDA) (2019). Appropriate age range for introduction of complementary feeding into an infant’s diet. EFSA J..

[26] van Stuijvenberg M., Eisses A.M., Grüber C., Mosca F., Arslanoglu S., Chirico G., Braegger C.P., Riedler J., Boehm G., Sauer P.J.J. (2011). Do prebiotics reduce the number of fever episodes in healthy children in their first year of life: A randomised controlled trial. Br. J. Nutr..

[27] Dumrongwongsiri O., Winichagoon P., Chongviriyaphan N., Suthutvoravut U., Grote V., Koletzko B. (2022). Zinc and iron adequacy and relative importance of zinc/iron storage and intakes among breastfed infants. Matern. Child Nutr..

[28] Adnan N.A., Breen E., Tan C.A., Wang C.C., Jalaludin M.Y., Lum L.C.S. (2024). Iron deficiency in healthy, term infants aged five months, in a pediatric outpatient clinic: A prospective study. BMC Pediatr..

[29] Sittimol A., Saengpanit P., Vatthana N., Rojmahamongkol P. (2024). Prevalence of iron deficiency anemia in exclusively breastfed infants after a 5-month iron supplementation. Sci. Rep..

[30] Ierodiakonou D., Garcia-Larsen V., Boyle R.J. (2017). Allergenic Food Introduction and Childhood Risk of Allergic or Autoimmune Disease-Reply. JAMA.

[31] De Silva D., Halken S., Singh C., Muraro A., Angier E., Arasi S., Arshad H., Beyer K., Boyle R., Du Toit G. (2020). Preventing food allergy in infancy and childhood: Systematic review of randomised controlled trials. Pediatr. Allergy Immunol..

[32] Skjerven H.O., Lie A., Vettukattil R., Rehbinder E.M., LeBlanc M., Asarnoj A., Carlsen K.-H., Despriee Å.W., Färdig M., Gerdin S.W. (2022). Early food intervention and skin emollients to prevent food allergy in young children (PreventADALL): A factorial, multicentre, cluster-randomised trial. Lancet.

[33] Perkin M.R., Logan K., Marrs T., Radulovic S., Craven J., Flohr C., Lack G., Young L., Offord V., DeSousa M. (2016). Enquiring About Tolerance (EAT) study: Feasibility of an early allergenic food introduction regimen. J. Allergy Clin. Immunol..

[34] Tanase M., Pistol A.M., Zmarandache D.D.D., Stanciu I.A., Munteanu A. (2026). Review Regarding the Impact of Breastfeeding on Early Childhood Caries. Children.

[35] Kersting M., Dulon M. (2002). Assessment of breast-feeding promotion in hospitals and follow-up survey of mother-infant pairs in Germany: The SuSe Study. Public Health Nutr..

[36] Rebhan B., Kohlhuber M., Schwegler U., Koletzko B. (2009). Infant feeding practices and associated factors through the first 9 months of life in Bavaria, Germany. J. Pediatr. Gastroenterol. Nutr..

[37] Avilla J.C., Giugliani C., Bizon A., Martins A.C.M., Senna A.F.K., Giugliani E.R.J. (2020). Association between maternal satisfaction with breastfeeding and postpartum depression symptoms. PLoS ONE.

[38] Rosenbaum D.L., Gillen M.M., Markey C.H. (2020). Feeling let down: An investigation of breastfeeding expectations, appreciation of body functionality, self-compassion, and depression symptoms. Appetite.

[39] Roth M.C., Humphreys K.L., King L.S., Gotlib I.H., Robakis T.K. (2021). Breastfeeding Difficulties Predict Mothers’ Bonding with Their Infants from Birth to Age Six Months. Matern. Child Health J..

[40] Rowles G., Keenan J., Wright N.J., Hughes K., Pearson R., Fawcett H., Braithwaite E.C. (2025). Investigating the impact of breastfeeding difficulties on maternal mental health. Sci. Rep..

[41] Wright E.A., Mehta A., Nelson A.L. (2025). Mothers with Breastfeeding Difficulty Report Increased Depressive Symptoms and Impaired Maternal-Infant Bonding on Social Media. J. Womens Health.

